# Associations between Circulating Markers of Cholesterol Homeostasis and Macrovascular Events among Patients Undergoing Hemodialysis

**DOI:** 10.3390/nu13031014

**Published:** 2021-03-21

**Authors:** Wen-Chin Lee, Wei-Hung Kuo, Sin-Hua Moi, Barry Chiu, Jin-Bor Chen, Cheng-Hong Yang

**Affiliations:** 1 Division of Nephrology, Department of Internal Medicine, Kaohsiung Chang Gung Memorial Hospital and Chang Gung University College of Medicine, Kaohsiung 83301, Taiwan; leewenchin@gmail.com (W.-C.L.); b8701144@cgmh.org.tw (W.-H.K.); 2 Center of Cancer Program Development, E-Da Cancer Hospital, I-Shou University, Kaohsiung 84001, Taiwan; moi9009@gmail.com; 3 Department of Epidemiology, Brown University School of Public Health, Providence, RI 02903, USA; barryprchiu@brown.edu; 4 Department of Electronic Engineering, National Kaohsiung University of Science and Technology, Kaohsiung 80778, Taiwan; chyang@cc.kuas.edu.tw; 5 Ph.D. Program in Biomedical Engineering, Kaohsiung Medical University, Kaohsiung 80708, Taiwan

**Keywords:** cholesterol homeostasis, hemodialysis, macrovascular events, mortality

## Abstract

Current strategies targeting serum cholesterol bring limited benefits to mortality and macrovascular events prevention among hemodialysis patients. Direct measurements and analysis on circulating markers of cholesterol homeostasis could be promising solutions to this bottleneck. We prospectively enrolled 90 maintenance hemodialysis patients and 9 healthy controls in 2019 for 1 year. We measured circulating desmosterol and lathosterol as markers for cholesterol synthesis and campesterol and sitosterol for cholesterol absorption. At baseline, hemodialysis patients showed higher levels of campesterol (*p* = 0.023) compared to healthy controls. During follow-up, we identified 14 (15.4%) patients who experienced macrovascular events. Comparisons of cholesterol homeostasis markers between cohorts with and without macrovascular events showed no significant differences in markers of cholesterol synthesis or absorption. Using logistic regression analysis, the odds ratio was not statistically significant for the prediction of macrovascular events after full-adjusting for age, sex, diabetes, serum albumin, cholesterol, and triglyceride. We concluded that hemodialysis patients demonstrated higher level of cholesterols absorption, indicated by circulating campesterol compared to healthy subjects. Markers for cholesterol homeostasis were not significantly associated with macrovascular events during a 1-year follow-up. Our results shed light on the novel therapeutic target of modulating cholesterol absorption in HD patients.

## 1. Introduction

Macrovascular events are common in chronic kidney disease (CKD) and hemodialysis (HD) populations [1,2,3]. These events are the most common causes of death among HD patients [4,5]. Dyslipidemia has long been recognized as a potentially modifiable risk factor in the prevention of macrovascular events in HD patients. However, randomized control trials on cholesterol-lowering drugs fail to show benefits for macrovascular events prevention in HD patients. In addition to current research on cholesterol levels among HD patients, investigations on the alterations of cholesterol homeostasis are crucial to better understand the link between dyslipidemia and macrovascular events in HD patients.

Cholesterol is an insoluble lipid molecule that is involved with the structure and function of cellular membrane bilayers and is a substrate for steroid hormone biosynthesis. A complex network is required to maintain intracellular cholesterol homeostasis in cholesterol biosynthesis, uptake, efflux, conversion, esterification, and trafficking [6,7]. Various cells have the capacity to regulate cellular free cholesterol concentrations through endogenous synthesis, intracellular esterification, and excretion [8]. Whole-body cholesterol content is regulated by balancing input (intestinal absorption of dietary and biliary cholesterol) and output (hepatic and extra-hepatic synthesis) [9,10]. Various circulating markers have been assigned to reflect endogenous cholesterol synthesis (lathosterol, desmosterol, mevalonate, and squalene), intestinal cholesterol absorption (sitosterol, campesterol, and cholestanol), or bile acid synthesis (7alpha-hydroxy-4-cholesten-3-one) in population study [11,12,13,14,15].

There has been limited research on cholesterol homeostasis in CKD and HD population. In an earlier study among dialysis patients with a small sample size, both plasma biomarkers of cholesterol intestinal absorption (campesterol) and endogenous cholesterol synthesis (lathosterol) were reduced compared to healthy participants [16]. Another study showed higher levels of cholesterol intestinal absorption markers (cholestanol) and lower levels of endogenous cholesterol synthesis markers (lathosterol) in HD patients when compared to healthy controls [17].

Considering the lack of a comprehensive assessment of cholesterol homeostasis in HD patients, we performed the present study to examine cholesterol homeostasis via direct measurement of circulating markers of cholesterol intestinal absorption and endogenous cholesterol synthesis. This quantification of circulating biomarkers for cholesterol homeostasis is warranted. The traditional lipid profile is usually derived rather than directly measured. In contrast, mass spectrometry-based cholesterol profiling can accurately measure circulating markers of cholesterol homeostasis. This approach allows researchers to detect alterations in various metabolic pathways that control cholesterol homeostasis. Furthermore, we assessed the association between circulating markers of cholesterol homeostasis and macrovascular events in HD patients.

## 2. Materials and Methods

### 2.1. Study Design and Participants 

Adult patients (>18 years) who underwent maintenance HD (4-h thrice weekly) for at least 3 months in the outpatient clinic in Kaohsiung Chang Gung Memorial Hospital in Taiwan from August 2019 to September 2019 were enrolled in this study. All participants underwent HD with appropriate dialyzers (Supplementary Table S1) [18] and bicarbonate-based dialysate, dialysate sodium 140 mEq/L, calcium 3.0 mEq/L, and potassium 2.0 mEq/L. Blood flow rate was 250–300 cc/min and dialysate flow rate was 500 cc/min. None of the participants received hemodiafiltration. The exclusion criteria were as follows: ongoing treatment for malignancy, acute inflammatory diseases, hospitalization within 3 months, malnutrition defined by serum albumin level <3.5 g/dL, pregnancy, and using lipid-lowering drug treatment at least 3 months prior to the study. All participants did not have pre-existing macrovascular events and were followed to September 2020. Healthy controls were recruited voluntarily in the outpatient clinic by posted protocol notification. Informative patient data, including demographic profiles, laboratory parameters, and macrovascular events in the study period were collected.

### 2.2. Definition and Assessment of Macrovascular Events

Macrovascular events were defined as occurrence of angina pectoris, unstable angina, myocardial infarction, cardiac catheterization-proved coronary artery insufficiency, cerebrovascular accident, or transient ischemic attack. Information of macrovascular events was confirmed through medical records from the study clinic and hospitalization records.

### 2.3. Biochemistry

All blood samples from HD participants in the fasting status and in mid-week (Wednesday and Thursday) were obtained. Blood samples for biochemistry measurement were obtained using commercial kits and an autoanalyzer (Hitachi 7600-210, Hitachi Ltd., Tokyo, Japan). Albumin levels were measured using the bromocresol green method. The plasma total cholesterol, triglyceride, and high-density lipoprotein cholesterol (HDL-C) levels were determined enzymatically on the Eroset Hitachi 7600-210 analyzer. The low-density lipoprotein cholesterol (LDL-C) levels were calculated using to the Friedewald formula [19], which provides reliable values up to a triglyceride level of 4.0 mmol/L.

### 2.4. Measurements of Circulating Markers of Cholesterol Homeostasis

Concentrations of desmosterol, lathosterol, campesterol, and sitosterol were quantified using a gas chromatography-mass spectrometry method similar to prior studies [20]. Briefly, plasma samples from HD patients and healthy controls were added with 10 uL 5-alpha-Cholestane in the standard solution, and potassium hydroxide standard for saponification for 1 h after centrifugation. After saponification, the solution was added with n-Hexane. Later, the derivatization reagents (BSTFA-TMCS = 99:1) were added to the residue. After the derivatization reaction, the samples were added with the standard solution according to the preparation of standard solution tables provided by the commercial company (Ardent BioMed LLC, Mt. View, CA, USA). Linear regression was used to quantitatively determine the individual standard curve according to internal quantitative ion area ratio (x) and compound concentration (y). Since the non-cholesterol sterols are transported in plasma by lipoproteins, their concentrations were expressed relative to the concentration of total cholesterol (mmol/mol of cholesterol) to correct for the differing number of lipoprotein acceptor particles. Cholesterol metabolic balance score was calculated as desmosterol + lathosterol/campesterol + sitosterol. We defined the cholesterol metabolic balance scores as follows: score >1.1, increased endogenous synthesis; 0.5–1.1, normal metabolism; and 0.2–0.5, increased intestinal absorption.

### 2.5. Statistical Analysis

Baseline demographic characteristics and laboratory measurements among HD patients and healthy controls are presented as frequency (percentage) and mean (standard deviation). The distribution difference was estimated using the independent two-sample *t*-test, Mann–Whitney, chi-square, or Fisher’s exact test. Logistic regression analysis was performed to evaluate the association between macrovascular events and baseline characteristics in HD patients. The correlation between the cholesterol absorption and synthesis parameters and associated variables was estimated using the Pearson correlation test. The cholesterol absorption and synthesis parameters, which included desmosterol, lathosterol, campesterol, sitosterol, and cholesterol metabolic balance scores, were included in the univariate logistic regression model. All included variables were retained in the fully adjusted logistic regression model. Demographic characteristics including age, sex, diabetes, albumin, cholesterol, and triglyceride were involved as covariates. All *p*-values were two-sided, and *p* < 0.05 was considered statistically significant. All statistical analyses were performed using the R 4.0.3 software (R Core Team, 2020 R Foundation for Statistical Computing, Vienna, Austria).

## 3. Results

**Figure 1:**
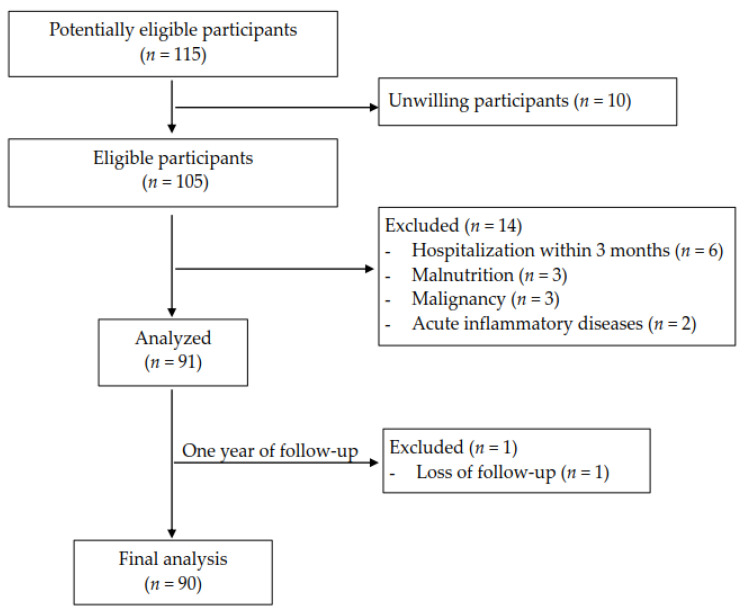
Study design and flowchart of patient selection.

We pre-screened potentially eligible participants (*n* = 115) who had received maintenance HD in outpatient clinic in August 2019. Ten patients expressed not to participate this study owing to individual reason. Thus, 105 HD patients entered our screening stage. After implementing our selection criteria, 91 patients were enrolled in the study. In a 1-year follow-up, 90 patients completed the study (Figure 1). Nine healthy volunteers were also recruited at the baseline period.

### 3.1. Characteristics of Circulating Markers of Cholesterol Homeostasis in HD Patients

Baseline characteristics in the study cohort are shown in the Table 1. The mean age was 65 years, and gender distribution was similar within the HD cohort. HD patients showed lower levels of desmosterol (*p* = 0.523) and lathosterol (*p* = 0.499) and higher levels of campesterol *(p* = 0.023) and sitosterol (*p* = 0.199) compared to healthy controls. Cholesterol metabolic balance scores were lower in HD cohort compared to healthy controls, though the difference was not statistically significant (*p* = 0.109). 

### 3.2. Differences between HD Patients with and without Macrovascular Events

Patients who had macrovascular events in the study period were significantly older in age (*p* < 0.001), had higher body mass index (*p* < 0.001), and longer dialysis vintage (*p* < 0.001) compared to those without events. Although patients with macrovascular events showed lower levels of intestinal cholesterol absorption, the differences did not reach statistical significance. Cholesterol metabolic balance scores were similar between two groups (Table 2).

### 3.3. Correlations of Cholesterol Homeostasis Markers

In correlation analysis, body mass index showed a significantly positive correlation between lathosterol and cholesterol metabolic balance score and negative between campesterol and sitosterol. The association between cholesterol metabolic balance score and cholesterol synthesis markers was significantly positive. Conversely, the association between cholesterol intestinal absorption markers was significantly negative. In addition, total cholesterol showed a significantly negative correlation with lathosterol. Triglyceride showed a significantly negative correlation with campesterol and sitosterol. Regarding lipoprotein profiles, HDL-C showed significantly positive correlation with sitosterol. LDL-C showed significantly negative correlation with lathosterol (Table 3). 

### 3.4. Predictions of Macrovascular Events in HD Patients by Cholesterol Homeostasis Markers

Results from unadjusted and adjusted models for macrovascular events prediction using logistic regression analysis are shown in Table 4. Overall, individual markers for cholesterol synthesis and absorption and cholesterol metabolic balance score did not show significant odds ratio (OR) for macrovascular events prediction. 

## 4. Discussion

Cholesterol homeostasis is complex in HD patients. Strategies simply to decrease LDL-C, as seen in the 4D (Die Deutsche Diabetes Dialyse Studie) and AURORA (A Study to Evaluate the Use of Rosuvastatin in Subjects on Regular Hemodialysis), failed to show a survival benefit in this population despite leading to an LDL cholesterol reduction in a magnitude shown to significantly reduce cardiovascular morbidity and mortality in the general population [21,22,23]. Cholesterol homeostasis involves hepatic and extra-hepatic endogenous synthesis and intestinal and biliary absorption of exogenous cholesterol. Animal-derived cholesterol and plant-derived phytosterols or plant sterols are the main component of dietary sterols. Among plant sterols, sitosterol and campesterol are most abundant. An observation from inherited disease patients with ABCG5 and/or ABCG8 deficiency presented sitosterolemia and premature coronary disease [24,25]. This phenotypical observation leads to a hypothesis that high levels of plant sterols are atherogenic. However, data from prior studies did not reveal consistent association between coronary heart disease and plant sterols. The risk of incident myocardial infarction in men increased 1.8-fold for sitosterol in the Prospective Cardiovascular Munster study [26]. The Framingham Offspring Study reported that campesterol, sitosterol, and cholestanol were significantly associated with cardiovascular disease [27]. Additionally, several small-scale studies reported that higher levels of cholesterol absorption markers increased the risk for cardiovascular diseases [28,29]. In contrast, the EPIC-Norfolk study and the Longitudinal Aging Study Amsterdam (LASA) study both reported that increased cholesterol absorption markers were associated with decreased risk for cardiovascular disease [30,31]. Moreover, a study reported no significant association between sitosterol and campesterol and coronary atherosclerosis among middle-aged men and women [32]. Recently, a study reported that a low serum lathosterol is correlated with an increased risk for cardiovascular events and an excess of all-cause mortality across 4.9 years of follow-up. None of the other cholesterol homeostasis markers demonstrated this relationship [33]. Various factors may account for the variability in the above studies, such as study design and selection bias, diverse participants in age, health, and genetic background, individual lipoprotein profile, gut microbiome, and variable experimental methods to measure concentration of plant sterols. Given the above observation, a standardization of experimental methods and large-scale longitudinal study is needed to clarify this controversial issue.

There are only a few studies that address cholesterol homeostasis and its relationship with cardiovascular risk within CKD or HD populations. CKD patients were reported to have impaired intestinal fat absorption [34]. In a study on eight HD patients, investigators reported that HD patients demonstrated significantly lower fractional cholesterol synthesis (lathosterol) and lower lathosterol to cholesterol ratio compared to healthy controls [16]. Investigators concluded that hepatic cholesterol synthesis was impaired in HD patients since the ratio of lathosterol to cholesterol reflecting hepatic activity of HMG-CoA reductase and fecal total hepatic cholesterol balance synthesis [16,35,36]. Later, a study reported that HD patients had lower lathosterol and higher cholestanol levels compared to healthy controls [17]. Results of our study were in line with previous studies. We found that HD patients demonstrated lower levels of cholesterol synthesis markers (lathosterol and desmosterol) and higher levels of cholesterol absorption markers (campesterol and sitosterol) compared to healthy controls. Our analysis using cholesterol metabolic balance scores also indicated increased cholesterol absorption in HD patients. These results reminded us of the cardiovascular protection effects shown within the SHARP (the Study of Heart and Renal Protection) study in which significant reduction in cardiovascular events in CKD and HD is achieved by combining inhibition of cholesterol synthesis via statin treatment and inhibition of intestinal cholesterol absorption via ezetimibe [37].

In the present study, we found that body mass index demonstrated a significantly positive correlation with lathosterol and a negative association with campesterol and sitosterol. Although the exact mechanisms were not clear, our results were partly supported from research on insulin resistance, metabolic syndrome, and diabetes [38,39,40,41,42]. Patients with these insulin resistance-mediated metabolic derangements exhibited a high cholesterol synthesis and low cholesterol absorption profile. Greater cholesterol synthesis in the liver would lead to less cholesterol absorption. Another interesting finding in our study was that lathosterol demonstrated significantly negative correlation with total cholesterol and LDL-C. On the contrary, campesterol and sitosterol demonstrated a positive correlation with HDL-C and a negative correlation with triglyceride. Considering a reciprocal relationship between cholesterol synthesis and absorption, it is reasonable to explain our correlation results. Notably, several food products have been added with plant sterols for their effects on reducing blood cholesterol level. This in turn reduces cholesterol absorption in the intestine. Consequently, blood levels of LDL-C are reduced because of foods enriched with plant sterols [43]. We did not collect dietary information of our participants. Therefore, the influence of dietary plant sterols on correlation analysis cannot be obtained in our statement.

Interestingly, the association between cholesterol homeostasis and mortality is rarely reported for CKD and HD populations. Rogacev et al. reported that cholestanol above median levels had a 2.24-fold risk for all-cause mortality across 3.4 years in HD patients. However, lower lathosterol levels did not demonstrate a similar relationship [17]. In our study, we also did not find the associations between cholesterol synthesis and absorption markers with macrovascular events after adjusting for potential confounding factors in our HD cohort. However, our results were limited by the small number of composite events and a short follow-up period. Furthermore, our study cohort presented reasonable control in their blood pressure, HDL-C, and LDL-C levels. A plausible explanation may be rooted in these favorable clinical situations. Non-traditional risk factors are known to play crucial roles in macrovascular events in HD patients [44,45], but the weights of impacts between these factors and cholesterol homeostasis markers remain unknown. An additional explanation for the lack of association between cholesterol homeostasis markers and macrovascular events in our study might be that non-traditional risk factors outweigh the cholesterol homeostasis markers in macrovascular events in HD patients. To overcome our limitations and to clarify these issues, a further large-scale study with thorough analysis of traditional and non-traditional risk factors and longer follow-up periods is required.

Our study has some limitations. First, the observational design does not completely exclude the possibility of potential confounders that influence the observed outcomes. Although some confounders such as age, blood pressure, and diabetes were adjusted in our study, we agree that some confounders may partly contribute to the composite outcomes. Second, our dataset lacked relevant information known to influence cholesterol homeostasis such as dietary records, insulin sensitivity assessment, specific genotypes, and genetic polymorphisms. Therefore, we cannot exclude the influence of these unmeasured factors. Third, we did not consider the variation in diurnal and time changes in cholesterol homeostasis. Although we used cholesterol metabolic balance scores to diminish the influence of these drawbacks, the validation of our methods warrants further testing through a large-scale population study. Fourth, our study cohort was predominantly of Asian ethnicity and may have a unique lipoprotein profile. Care must be taken when our results are extrapolated to other ethnicities and disease states. Finally, the number of participants in our study was relatively small and composite events were also small. Furthermore, the follow-up duration was only 1 year. These considerations may diminish statistical power in our results. However, our study remains strength in understanding the impact of cholesterol homeostasis on macrovascular events in HD population. To our knowledge, our study is the first report of the relationship between cholesterol homeostasis and macrovascular events in an Asian HD population. In addition, our study could be a proxy to further investigate the diverse cholesterol homeostasis profiles among dialysis populations. Therefore, effective therapeutic measures could be possible to prevent vascular events in HD populations by considering cholesterol absorption and synthesis profiles.

## 5. Conclusions

In conclusion, our study demonstrated increased circulating campesterol and a trend of increased cholesterol intestinal absorption and reduced cholesterol synthesis in HD patients. Although this cholesterol homeostasis pattern was not directly associated with macrovascular events across a 1-year observational period, it sheds light on the novel therapeutic target of modulating cholesterol absorption in HD patients.

## Figures and Tables

**Table 1:** Baseline characteristics in hemodialysis (HD) patients and healthy controls (*n* = 99).

Variables	HD (*n* = 90)	Healthy Control (*n* = 9)	*p*
Age, years	65 ± 7	62 ± 11	0.196
Sex			1.000
Female	45 (50.0%)	4 (44.4%)	
Male	45 (50.0%)	5 (55.6%)	
BMI, kg/m^2^	22.6 ± 4.0	22.1 ± 3.1	0.766
Systolic blood pressure, mmHg	137 ± 30	N/A	
Diastolic blood pressure, mmHg	67 ± 14	N/A	
Dialysis vintage, years	10.4 ± 7.49	N/A	–
Diabetes	16 (17.8%)	0 (0 %)	–
Etiologies of renal failure			–
Primary kidney disease	37 (41.1%)	N/A	
Systemic disease	37 (41.1%)	N/A	
Others	16 (17.8%)	N/A	
Desmosterol, 10^2^ mmol/mol cholesterol	61.6 ± 86.5	65.2 ± 39.9	0.523
Lathosterol, 10^2^ mmol/mol cholesterol	111.6 ± 95.9	157.2 ± 136.3	0.499
Campesterol, 10^2^ mmol/mol cholesterol	330.0 ± 179.5	207.9 ± 91.4	0.023
Sitosterol, 10^2^ mmol/mol cholesterol	218.4 ± 128.4	168.3 ± 89.0	0.199
Cholesterol metabolic balance score	0.4 ± 0.3	0.7 ± 0.6	0.109
Laboratory measurements			
Hemoglobin, g/dL	10.7 ± 1.3	13.5 ± 1.9	<0.001
Albumin, g/dL	3.9 ± 0.3	4.3 ± 0.3	0.015
BUN, mg/dL *	69.0 (57.0–83.0)	14.0 (13.0–16.0)	<0.001
Cr, mg/dL	10.4 ± 2.1	1.0 ± 0.4	<0.001
Total Cholesterol, mg/dL *	153.5 (136.0–183.0)	191.0 (177.0–199.0)	0.005
Triglyceride, mg/dL *	114.5 (84.0 –165.0)	117.0 (84.0–131.0)	0.789
HDL-C, mg/dL	45.4 ± 15.3	52.1 ± 9.6	0.049
LDL-C, mg/dL	90.2 ± 34.6	122.3 ± 41.1	0.005

Abbreviations: HD—hemodialysis; BMI—body mass index; BUN—blood urea nitrogen; Cr—creatinine; HDL—high-density lipoprotein; LDL—low-density lipoprotein; C—cholesterol; * median (interquartile range).

**Table 2:** Baseline characteristics in HD patients with and without macrovascular events (*n* = 90).

Variables	With Events (*n* = 14)	Without Events (*n* = 76)	*p*
Age, years	69 ± 8	64 ± 6	<0.001
Sex			0.144
Female	4 (28.6%)	41 (53.9%)	
Male	10 (71.4%)	35 (46.1%)	
BMI, kg/m^2^	24.0 ± 6.0	22.3 ± 3.3	<0.001
Systolic blood pressure, mmHg	136 ± 30	154 ± 24	0.094
Diastolic blood pressure, mmHg	67 ± 15	68 ± 10	0.528
Dialysis vintage, years	11.4 ± 9.1	10.2 ± 7.2	<0.001
Diabetes	3 (21.4%)	13 (17.1%)	0.709
Desmosterol, 10^2^ mmol/mol cholesterol	61.7 ± 49.6	61.5 ± 91.9	0.925
Lathosterol, 10^2^ mmol/mol cholesterol	99.1 ± 108.0	113.9 ± 94.1	0.214
Campesterol, 10^2^ mmol/mol cholesterol	298.1 ± 139.7	335.9 ± 186.0	0.652
Sitosterol, 10^2^ mmol/mol cholesterol	199.2 ± 83.5	221.9 ± 135.2	0.854
Cholesterol metabolic balance score	0.4 ± 0.4	0.4 ± 0.3	0.544
Laboratory measurements			
Hemoglobin, g/dL	10.7 ± 1.3	10.7 ± 1.3	0.982
Albumin, g/dL	3.9 ± 0.4	3.9 ± 0.3	0.551
BUN, mg/dL	75.0 (64.0–84.0)	68.0 (56.0–80.0)	0.319
Cr, mg/dL	10.5 ± 2.3	10.4 ± 2.0	0.555
Total Cholesterol, mg/dL *	141.5 (137.0–164.0)	158.0 (136.0–186)	0.157
Triglyceride, mg/dL *	110.0 (100.0–136.0)	117.0 (82.0–167.0)	0.925
HDL-C, mg/dL	39.9 ± 11.7	46.4 ± 15.7	0.148
LDL-C, mg/dL	81.1 ± 20.0	91.9 ± 36.6	0.395

Abbreviations: BMI—body mass index; BUN—blood urea nitrogen; Cr—creatinine; HDL—high-density lipoprotein; LDL—low-density lipoprotein; C—cholesterol; * median (interquartile range).

**Table 3:** Pearson correlation analysis between studied continuous variables.

Variables	Desmosterol	Lathosterol	Campesterol	Sitosterol	Cholesterol Metabolic Balance Score
Age	−0.15	−0.18	−0.16	−0.13	−0.08
BMI	−0.07	0.32 **	−0.21 *	−0.30 **	0.33 **
Desmosterol	–	0.40 ****	0.18	0.12	0.42 ****
Lathosterol	0.40 ****	–	0.07	−0.02	0.65 ****
Campesterol	0.18	0.07	–	0.85 ****	−0.40 ***
Sitosterol	0.12	−0.02	0.85 ****	–	−0.42 ****
Cholesterol metabolic balance score	0.42 ****	0.65 ****	−0.40 ***	−0.42 ****	–
Albumin	−0.07	0.14	−0.11	−0.03	0.08
Total Cholesterol	−0.17	−0.23 *	−0.18	−0.16	−0.11
Triglyceride	−0.18	0.06	−0.28 **	−0.32 **	0.13
HDL-C	0.15	−0.14	0.18	0.22 *	−0.18
LDL-C	−0.19	−0.23 *	−0.16	−0.14	−0.11

* *p* < 0.05, ** *p* < 0.01, *** p < 0.001, **** *p* < 0.0001.

**Table 4:** Logistic regression for macrovascular events prediction using cholesterol homeostasis markers.

Variables	Unadjusted		Full-Adjusted	
	OR (95% CI)	*p*	OR (95% CI)	*p*
Desmosterol	1.000 (0.989–1.006)	0.994	0.997 (0.968–1.008)	0.699
Lathosterol	0.998 (0.989–1.004)	0.598	0.997 (0.979–1.011)	0.697
Campesterol	0.999 (0.995–1.002)	0.470	1.000 (0.988–1.011)	0.939
Sitosterol	0.998 (0.992–1.003)	0.544	1.001 (0.985–1.015)	0.898
Cholesterol metabolic balance score	1.012 (0.132–4.993)	0.989	9.003 (0.013–4621.542)	0.467

Full-adjusted model includes age, sex, diabetes, albumin, cholesterol, and triglyceride as covariates.

## Data Availability

The data presented in this study are available on request from the corresponding author.

## Supplementary Materials

The following are available online at https://www.mdpi.com/2072-6643/13/3/1014/s1, Table S1: Distribution of dialyzers and diabetes in HD cohort (*n* = 90).
