# Supplementary material for: Associations between Circulating Markers of Cholesterol Homeostasis and Macrovascular Events among Patients Undergoing Hemodialysis
Source: Nutrients. 2021 Mar 21;13(3):1014. doi: 10.3390/nu13031014 (PMC8004048; doi:10.3390/nu13031014)
Supplement: Supplementary file 1 [file nutrients-13-01014-s001.pdf]

Supplementary Table S1. Distribution of dialyzers and diabetes in HD cohort ($n = 90$).

	With events ($n = 14$)	Without events ($n = 76$)
Components of Dialyzers (Membrane surface area)		
Polysulfone		
FX CorDiax 60 (1.4 m^2)	1	4
HF80s (1.8 m^2)	0	1
PS-2.0W (2.0 m^2)	0	1
PS-2.3W (2.3 m^2)	0	0
FX CorDiax 1000 (2.3 m^2)	1	3
HdF100S (2.4 m^2)	1	3
Cellulose triacetate		
FB-170U (1.7 m^2)	2	7
FB-210U (2.1 m^2)	1	15
Polyethersulfone		
EL-21H (2.1 m^2)	1	17
EL-25H (2.5 m^2)	4	17
Polymethylmethacrylate		
BG2.1U (2.1 m^2)	3	8
DM	3	13
Non-DM	11	63
